# Drug Information Unit, Medical Faculty of Novi Sad

**DOI:** 10.1186/2050-6511-13-S1-A10

**Published:** 2012-09-17

**Authors:** Saša Vukmirović, Ana Sabo, Zdenko Tomić, Nebojša Stilinović, Aleksandar Rašković, Boris Milijašević, Olga Horvat

**Affiliations:** 1 Department of Pharmacology, Toxicology and Clinical Pharmacology, Faculty of Medicine, University of Novi Sad, 21000 Novi Sad, Serbia

## Background

In Serbia in general there are several ways to obtain necessary information on various drugs. Medical and pharmaceutical professionals gather information from the National Agency of Drugs and Medical Devices or from various publications such as British National Fromulary, Physicians Drug Reference etc. The general population can obtain information from their general practicioner (GP) or pharmacist. At the Department of Pharmacology and Clinical Pharmacology, Medical Faculty of Novi Sad, there exists a Drug Information Unit, a regional center offering drug information to both professionals and the general population in Vojvodina (approximately 1,600,000 inhabitants).

## Methods

The client can require information by phone (more than 99.5% of all requests) or by e-mail. Interns in Clinical Pharmacology collect necessary data regarding the therapeutic problem (age and sex of the patient, other drugs taken, present diseases etc.). After solving the problem using the electronic databases or hardcopy sources, and upon the approval from the senior clinical pharmacologist, interns deliver the information to the client (both by phone and e-mail).

## Results

About 3% of all requests are from the general population (usually questions on interactions, side effects, dosing and administration); the remaining requests are from health (20% from GPs, and 80% from specialists) or pharmaceutical professionals. Almost 30% of all of the requests of the health professionals are regarding possible drug–drug or drug–disease interactions. About 12% of requests are related to side effects of the administered drug. Pregnancy and lactation are subjects of interest in 15% of the overall number of requests. Maximal doses allowed and posology have a share of about 11%. The remaining information is concerning the pharmacokinetics of the drugs, first line drugs for certain diseases, dosing in children etc.

## Conclusions

It can be concluded that the Drug Information Unit is a useful source of information for both professionals and the general population offering various information on different topics related to drugs.

